# An attention-guided dense convolutional network for automated diabetic foot ulcer segmentation

**DOI:** 10.3389/fendo.2026.1940557

**Published:** 2026-10-16

**Authors:** Rampriya R. S., Sabari Nathan, Arumuga Arun R.

**Affiliations:** 1 School of Computer Science Engineering and Information Systems, Vellore Institute of Technology, Vellore, Tamil Nadu, India; 2 Deloitte Tohmatsu DeepSquare Co., Ltd., Tokyo, Japan; 3 School of Computer Science and Engineering, Vellore Institute of Technology, Vellore, Tamil Nadu, India

**Keywords:** attention mechanism, deep learning, diabetic foot ulcer, medical image segmentation, multi-scale feature learning

## Abstract

**Introduction:**

A large number of diabetic patients have diabetic foot ulcers (DFU), and it is essential to segment the regions of the ulcer in a timely and accurate manner to treat them. The segmentation task is very difficult due to the highly irregular shape of the ulcers and very small areas of lesions, up to 0.04% of the DFU image.

**Methods:**

We aim to develop an attention-guided dense convolutional network, called UlcerNet, which is able to extract fine-grained ulcer features at different scales. It introduces dense convolutions, multi-scale residual attention blocks (MRADB), and modified receptive-field modules (MRFB) to enhance feature propagation and context modeling. We use the public DFUC 2022 dataset that contains 4000 close-up foot images to train and evaluate the UlcerNet model.

**Results:**

Our model is able to perform a Dice of 73.50% and mIoU of 76.04%, which gives better performance than the standard segmentation baselines (FCN8 49.93%, SegNet 47.68%, DeepLabV3 + 62.77%) and recent DFU segmentation baselines (TranUNet 42.61%, HarDNet-DFUS 59.81%, UFOS-Net 73.09%).

**Discussion:**

An ablation study further shows that each component, such as dense blocks, channel attention, spatial attention, MRADB, and MRFB, is significant for the accuracy. These findings show that UlcerNet offers good and precise DFU segmentation, which can be useful for clinical settings in the real-world.

## 1 Introduction

The most recent International Diabetes Federation (IDF) report (1) shows that in 2024, about 590 million individuals were living with diabetes in the world. This is expected to rise to 853 million by 2050. Nearly 240 million people with diabetes are currently undiagnosed, which represents approximately 44% of all cases. Another 541 million adults are facing a high risk of developing diabetes. More than 1.2 million children and teenagers have been diagnosed with it worldwide. Diabetes caused about 6.7 million deaths in 2021. High blood sugar during pregnancy is also a common condition that affects one in six newborns. It means nearly 21 million babies have high glucose levels before their birth.

According to national survey data, diabetes has increased from 5.5% (1990) to 11.4% in India (2). Thus, it leads to serious complications such as the possibility of limb amputation. In clinical practice, segmentation of DFUs from images is an important step in accurately monitoring ulcer size and healing progress. However, automatic DFU segmentation is very challenging: ulcers have irregular contours, can be extremely small (as little as 0.04% of the image area), and often have low-contrast boundaries relative to the surrounding skin. Standard image segmentation techniques often fail on DFUs due to these factors. In clinical practice, it is important to segment DFUs accurately in order to monitor their size and healing process. Automatic DFU segmentation is quite difficult, however, because the ulcer contours are irregular; the ulcers can be very small (as small as 0.04% of the image area), and the boundary between the ulcer and surrounding skin can have low contrast. These factors make traditional image segmentation methods inapplicable to DFUs.

Studies (3–5) show that nearly 70% of foot ulcers return even after treatment in at least 10% of DFU patients. Even though DFU appears as a physical wound, it also affects the patient’s mental health. Hence, it leads to stress, anxiety, and depression (6). In the recent literature, several automated methods, including machine learning (ML), computer vision, and deep learning (DL), have been introduced to perform wound segmentation. Recent DFU segmentation studies on convolutional neural network (CNN) based techniques show better performance results in comparison to others (7). Many conventional image analysis techniques have also been utilized for DFU wound segmentation (8, 9). Many studies stated that diabetic foot ulcers can be prevented once they are identified and treated early. However, healing gets delayed due to complications, such as infection (10). Longer treatment raises the chances of developing severe infections and increases amputation risk (11). In addition, manual measurement of ulcer wound area often leads to inaccurate results in achieving a precise clinical test. To address the above-mentioned issues, a robust automated system is required to detect and monitor DFU-related infections.

Several systems for medical telecommunication, such as systems for monitoring diabetes, have been established (12). The goals of these systems are to lower the price of healthcare facilities, improve access to medical facilities, enhance the quality of the current healthcare systems, and provide automated solutions for the shortage of specialists (13). Telecare systems have led to a significant change in the treatment of DFU, as specialized care can now be accessed even in rural settings. The viability of this has been demonstrated as a cloud-based deep learning solution (14) that implements the computational load shift onto mobile devices and thereby guarantees the provision of high-performance diagnosis using deep learning with hardware utilized in the local region. On this basis, Sendilraj et al. (15) introduced DFUCare, which is a system that integrates both detection and longitudinal care to provide healthcare professionals with a holistic digital history of healing. It is also important in the human and operational aspects of these systems.

The change from the old methods of clinical analysis to automated deep learning models has significantly enhanced the accuracy of the DFU diagnosis. Nonetheless, as stated in the core review by Zhang et al. (16), the problem of irregular morphology of ulcers persists, even though CNNs have replaced traditional machine learning models. Moreover, Alhasson and Alharbi (17) have discovered that irregular morphologies are best fit with deep learning. Low-contrast lesion boundaries are unable to be addressed using traditional models. In a deeper look into this paradigm, Alkhalefah et al. (18) studied using Generative AI (GenAI) to enhance accuracy, which is not a minor issue in the given area; still, the question of dividing small lesion areas remains a significant concern. Various deep learning techniques (32), light-weight architectures (33), and model optimization methods (34) are being investigated to enable intelligent and efficient analysis of different medical imaging applications such as diabetic foot ulcer (DFU) assessment.

In recent years, there has been substantial progress in the field of medical image segmentation with the development of deep learning. Specifically, biomedical applications extensively use encoder–decoder networks such as UNet and DeepLabV3+. Attention mechanisms such as Attention UNet and CBAM also improve these networks by selectively highlighting important spatial and channel information. Dense connectivity (inspired by DenseNet) enhances feature reuse and gradient flow. We integrate these concepts and develop an architecture tailored for the segmentation of DFU. The following key contributions were made in this work:

- In this work, we propose UlcerNet, which is a novel architecture of a dense CNN for DFU segmentation. Combines multi-scale feature extraction and hierarchical attention in a single network.
- To improve the extraction of fine boundaries of ulcers, we propose the Multi-Scale Residual Attention Dense Block (MRADB) by combining channel attention, spatial attention (CBAM), and residual dense connections.
- We design a Modified Receptive Field Block (MRFB) to collect multi-scale context around ulcers, making it more robust to size variations.
- The extensive experiments on the DFUC 2022 dataset prove that UlcerNet achieves superior performance over strong baselines and ablation variants. These results highlight UlcerNet’s promise for reliable DFU segmentation in clinical settings.

This research is structured in the following manner. Section 2 covers the past research that has been applicable to diabetic foot ulcer segmentation. Section 3 provides the suggested UlcerNet framework, its architecture, and its methodology. Section 4 is about the results analysis, the dataset, and the experiment architecture. Section 5 brings out the conclusion of the DFU results. Section 6 concludes the research work and gives recommendations for further research.

## 2 Materials and methods

This section gives the characteristics of the dataset and the proposed UlcerNet architecture for diabetic foot ulcer segmentation. The architecture combines new convolutional modules, attention mechanisms, and loss functions to achieve successful learning of features and an accurate definition of regions of ulcers.

### 2.1 Characteristics of the dataset

The data of DFUC 2022 is the basis of the experimental assessment in the proposed work. **Figure 1** displays instances of DFU images with their ground truth segmentation masks. It highlights the variability in ulcer shapes and sizes. It is created to offer difficult and heterogeneous annotated medical images to create and test automated segmentation algorithms. The dataset consists of high-resolution images of foot ulcers and their authoritative ground truth mask produced under the supervision of medical practitioners and experts in the field, with the objective of strict training and testing of deep learning models.

**Figure 1:**
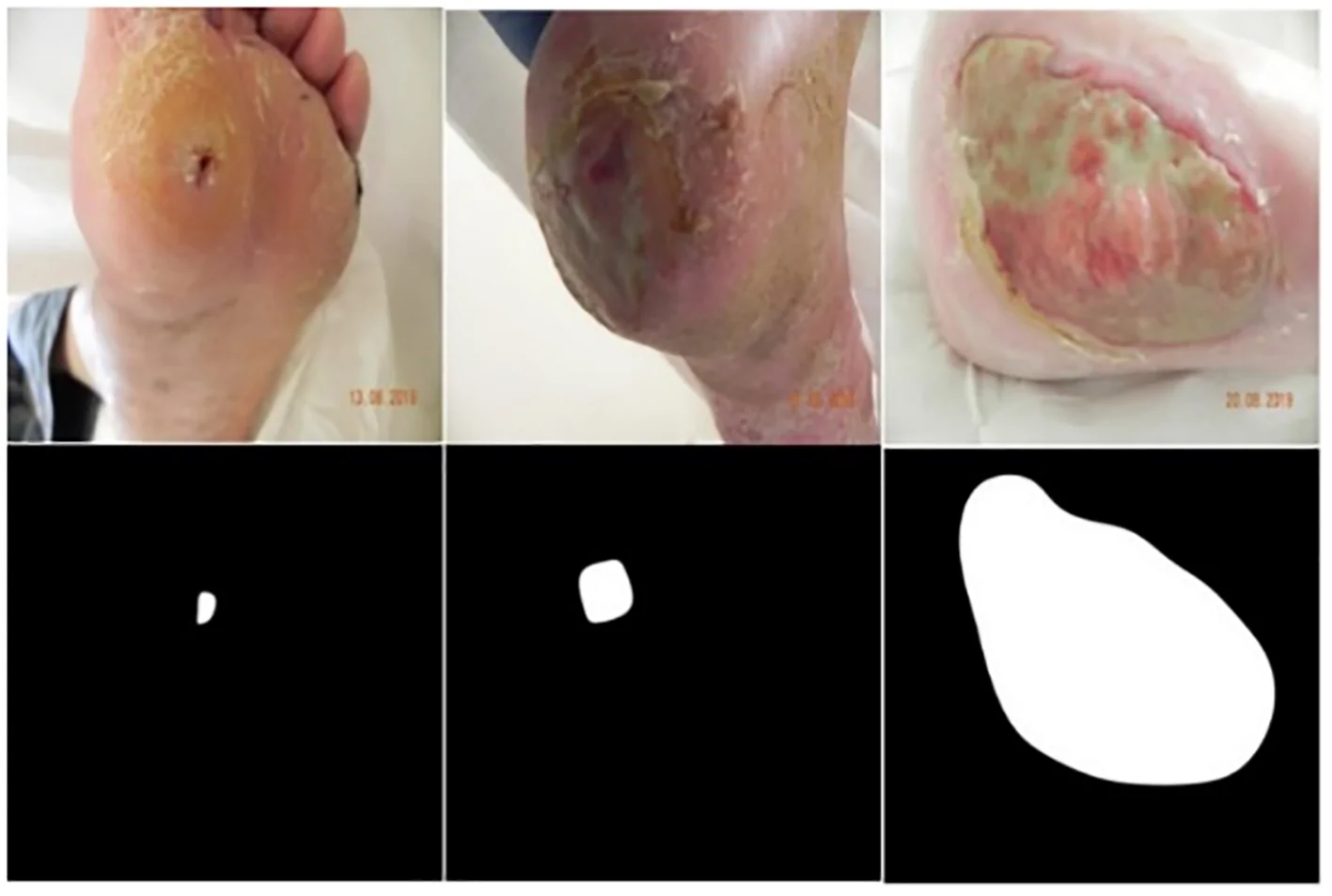
Real diabetic foot ulcer examples and ground truths of their segregations.

The publicly released DFUC2022 training release was used for this study, consisting of 1,620 training and 379 testing images (about 80%/20%) (19). The full DFUC2022 corpus has 4000 images, but there are no publicly available ground truth masks for the validation and test sets. This means that only images with annotations were used for supervised learning and quantitative assessment, which resulted in 1,999 annotated images.

The resolution of each image is 640×480. The morphology of the areas where the ulceration occurs is crucial for variability. This region ranges from a small size of 0.04% to a large size of 35.05% of the image size. It challenges the clinical diversity issues that are realistic. Such a dataset gives a solid background to work with and to compare segmentation models. It results in high accuracy and can be generalized to real-world applications of diabetic foot care. The dataset, which is utilized in this work, has a detailed description and is presented in **Table 1**.

**Table 1:** Aspects of the DFU dataset utilized in the proposed work.

Property	Value
Dataset	DFUC 2022
Training Images	1620
Testing Images	379
Resolution	640 × 480
Minimum Area of Ulcer	0.04% of the entire image size
Biggest Area of Ulcer	35.05% of the whole image size

The area of ulcers is expressed as a percentage of the entire image area.

### 2.2 Proposed methodology

The architecture of the proposed UlcerNet deep learning neural network is shown in **Figure 2**. The network starts with an input branch where a single multi-channel image of size 384 × 384 × 3 is taken. Here, it is first taken to a Coordinate Convolution Layer in order to add explicit spatial information. Next is a folding layer that is used to further compress the spatial size and deepens the channel to give a 96 × 96 × 80 feature map. The hierarchical features are then generated using MRADB, and the extraction of the encoder path is followed by downsampling of the encoder path through convolutional operations and max pooling.

**Figure 2:**
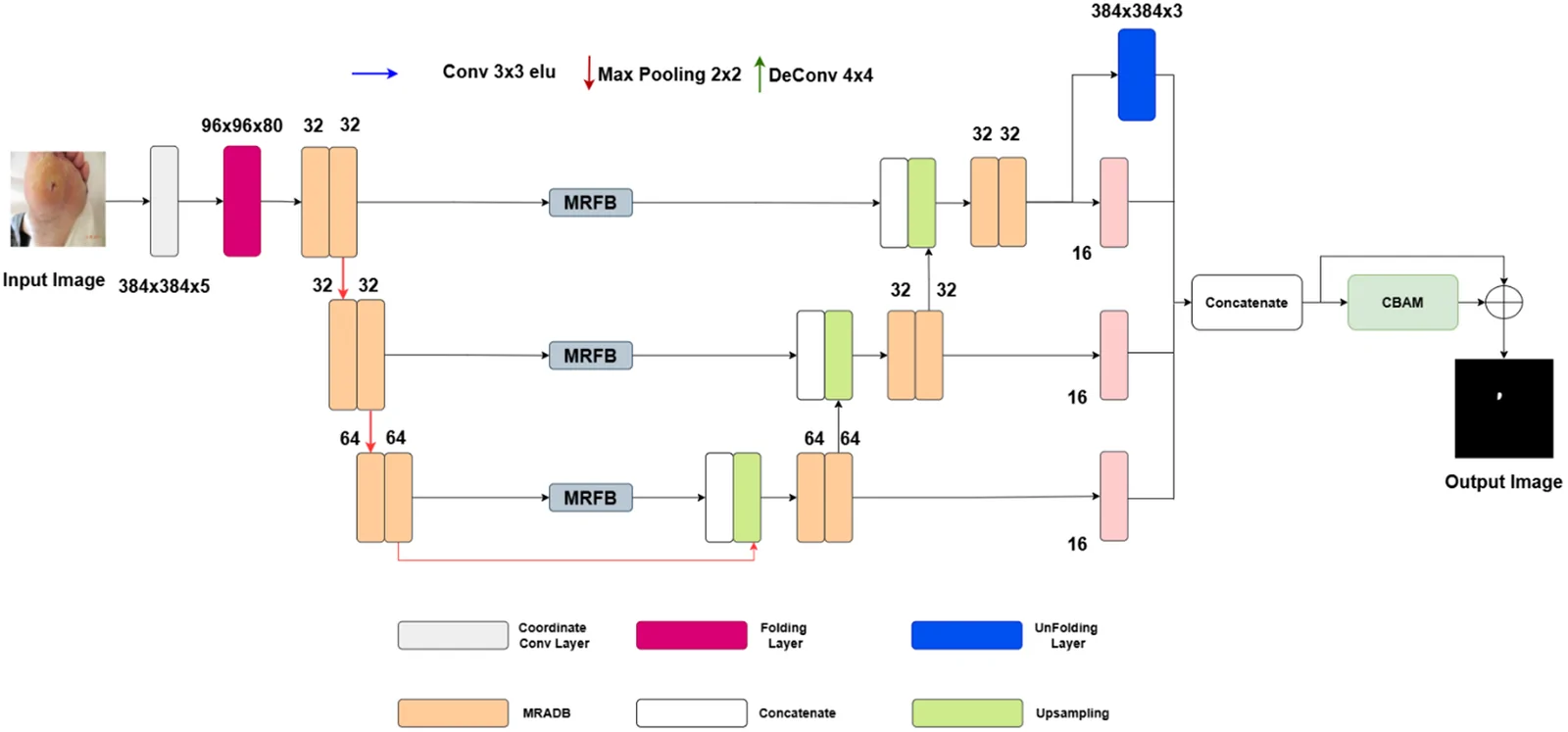
Overall architecture of the proposed Ulcer-Net model.

On every level, MRFBs are used to add feature representations that help characterize multi-scale context. The decoder path is a reflection of the encoder, based on the deconvolution layers to upsample and pre-incorporate skip connections to combine encoder features that are trained using further MRADBs. Three parallel outputs of shape 384 × 384 × 3 are created after reconstruction and input to an unfolding layer, and fused. A CBAM is used to further refine spatial and channel attention, and a residual connection combines the attention-refined output with the original unfolded feature to attain the ultimate segmentation map. The combination of spatial encoding and multi-scale feature extraction with attention mechanisms makes UlcerNet reliable and accurate in the segmentation of medical imaging, especially in complex medical imaging.

(1) $${\text{Z}}_{\text{ms}}={\text{ψ}}_{\text{attn}}(\sum_{\text{i}=1}^{\text{K}}{\text{g}}_{\text{i}}({\text{Z}}_{\text{in}})+{\text{Z}}_{\text{in}})$$

In Equation 1, Z_in_ denotes the input features to the multi-scale residual attention unit. The operator g_i_() represents the convolutional transformation applied at the i-th scale, where i = 1, 2, …, K. The summation aggregates multi-scale features extracted across *K* different convolutional paths. The function ${\text{ψ}}_{\text{attn}}()$ denotes the attention refinement module employed on the combined features. The final output Z_ms_ is the multi-scale, attention-refined feature map used for subsequent segmentation processing.

**Table 2** presents the network structure of the suggested UlcerNet architecture that processes input using coordinate folding and MSRA layers. A bottleneck MRFB captures deep features, followed by a decoder with upsampling and skip connections, which are used for spatial recovery. CBAM attention enhances the final feature map before projecting it into a two-class segmentation output.

**Table 2:** Network structure of the UlcerNet model.

Stage	Filter size	Stride	Output size
Input	–	–	384 × 384 × 3
Coordinate Convolution Layer	–	–	384 × 384 × 5
Folding Layer	–	–	96 × 96 × 80
Initial Conv + ELU	3 × 3/32	1 × 1	96 × 96 × 32
Layer 1 of MRADB	3 × 3/32	1 × 1	96 × 96 × 32
Max Pooling	2 × 2	2 × 2	48 × 48 × 32
Layer 2 of MRADB	3 × 3/64	1 × 1	48 × 48 × 64
Max Pooling	2 × 2	2 × 2	24 × 24 × 64
Layer 3 of MRADB	3 × 3/64	1 × 1	24 × 24 × 64
MRFB (Bottleneck)	3 × 3/64	1 × 1	24 × 24 × 64
Upsampling + Concat 1	4 × 4/64	2 × 2	48 × 48 × 64
Upsampling + Concat 2	4 × 4/32	2 × 2	96 × 96 × 32
Final Upsampling to Full Resolution	4 × 4/16	4 × 4	384 × 384 × 16
Fuse with CoordConv (Concat)	–	–	384 × 384 × 21
CBAM Attention	1 × 1/21	1 × 1	384 × 384 × 21
Final Projection (Unfold Layer)	1 × 1/2	1 × 1	384 × 384 × 2

#### 2.2.1 Modified receptive field block (MRFB)

MRFB is a special convolution module in the proposed U-Net architecture. It improves the multi-scale feature representation and contextual information extraction. The MRFB process starts with a 1×1 convolution for dimensionality reduction, followed by three parallel elements. Each element is made up of a 1×1 convolution with larger convolutions (3×3, 5×5, or 7×7), and a 3×3 dilated convolution. The 3×3 dilated convolution has different dilation rates, such as 1, 3, and 5. The outputs from all the elements are concatenated through a 1×1 convolution with ReLU activation. It facilitates effective gradient flow and stable learning. Such an organization improves contextual knowledge of the multi-scale and semantic attribute diffusion. It particularly proves useful in tasks where detailing is required, such as medical imaging.

In total, MRFB is employed in order to find specific localization and rich contextual awareness. It improves contextual feature extraction by means of hierarchical attention. **Figure 3** shows how the Modified Receptive Field Block (MRFB) is designed. It boosts contextual feature extraction by a hierarchical attention process.

**Figure 3:**
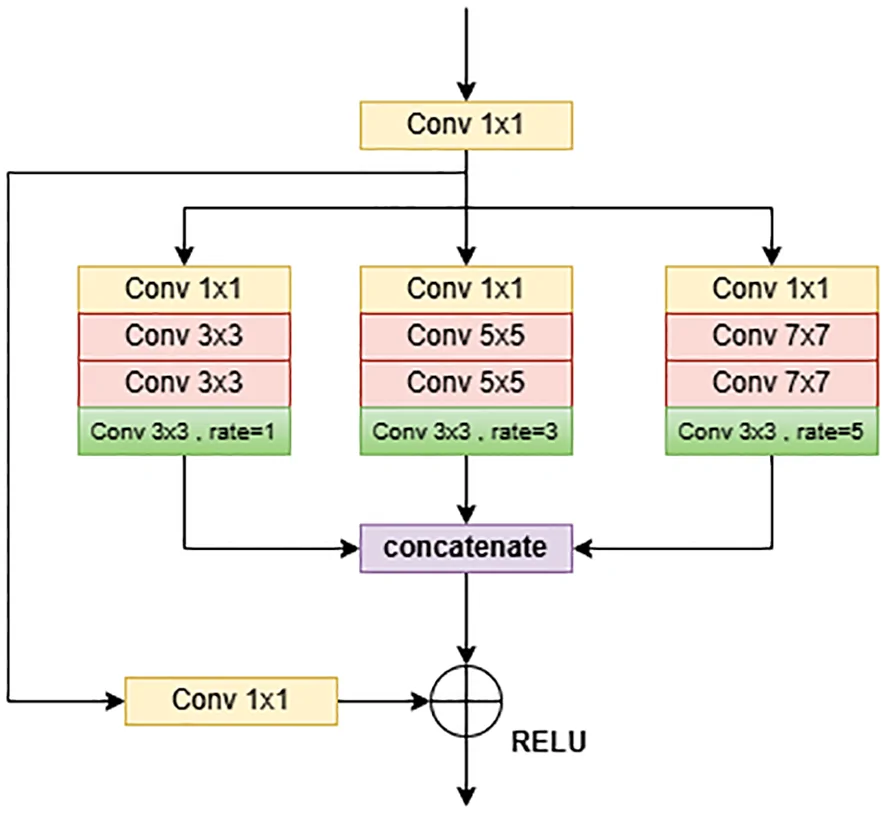
Design of modified receptive field block (MRFB).

(2) $${\text{F}}_{\text{MFRB}}=\text{σ}(\text{χ}+\text{h}([{\text{f}}_{1}(\text{χ}),{\text{f}}_{3}(\text{χ}),{\text{f}}_{5}(\text{χ})]))$$

The output feature map of the Modified Receptive Field Block is denoted by F_MRFB_ in Equation 2. χ denotes the feature map of the input. As f_1_(), f_3_() and f_5_() are functions, parallel convolutional paths are used with different kernel sizes and dilation settings to extract features based on different spatial sizes. The notation [] denotes channel-wise concatenation of the outputs of these branches, which are then fused with a 1×1 convolution denoted by h().

A residual connection was then used between the original input and the fused representation. Subsequently, it has a non-linear activation function, σ() which is usually the ReLU function, to ensure stable learning and feature extraction.

#### 2.2.2 Multi-scale residual attention dense block (MRADB)

MRADB is a new convolutional block that is used to boost the feature extractor with a hybrid of multi-scale convolution, dense connections, channel attention, and residual learning. The process begins with the application of a convolutional layer on the input feature map X_in,_ followed by a process of batch normalization and an activation using a non-linear function, usually LeakyReLU. **Figure 4** shows the MRADB, which depicts its architecture for capturing features at different scales with an in-built attention refinement.

**Figure 4:**
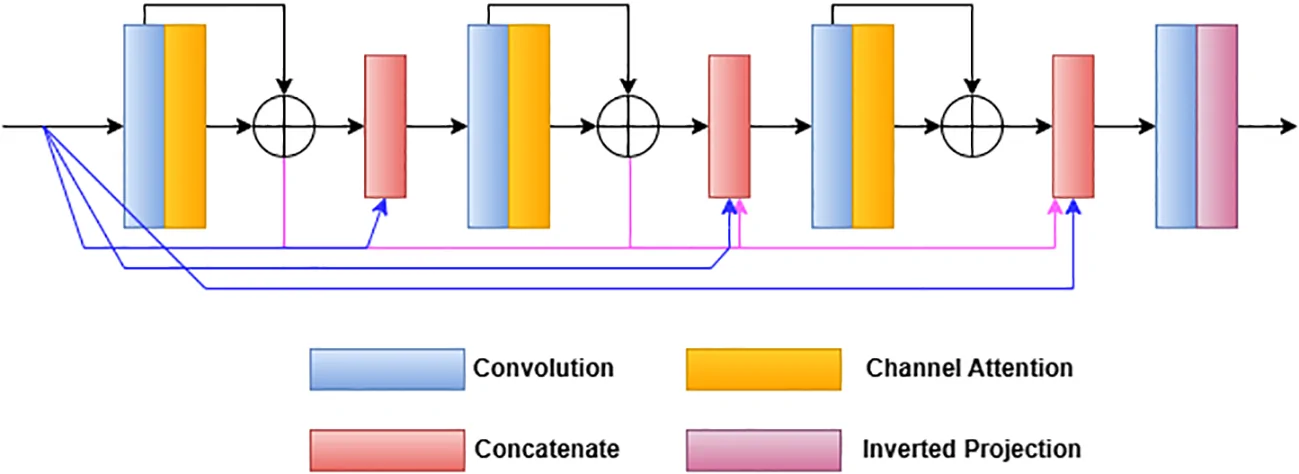
Multi-scale residual attention dense block (MRADB).

(3) $${\text{X}}_{0}=\text{BatchNorm }(\text{Activ }(\text{Conv }({\text{X}}_{\text{in}},3\times 3)))$$

In Equation 3, X_0_ represents the output of the initial convolution. Next, dense feature propagation is achieved by concatenating outputs from all previous layers, which is described in Equation 4:

(4) $${\text{F}}_{1\times 1}^{\text{att}}=\text{A}({\text{F}}_{1\times 1}),{\text{F}}_{3\times 3}^{\text{att}}=\text{A}({\text{F}}_{3\times 3}),{\text{F}}_{5\times 5}^{\text{att}}=\text{A}({\text{F}}_{5\times 5})$$

In Equation 5, original features are preserved and promote gradient flow. Each residual branch is self-gated by channel attention and added to itself:

(5) $${\text{R}}_{1\times 1}={\text{F}}_{1\times 1}^{\text{att}}+{\text{R}}_{1\times 1},{\text{R}}_{3\times 3}={\text{F}}_{3\times 3}^{\text{att}}+{\text{R}}_{3\times 3},{\text{R}}_{5\times 5}={\text{F}}_{5\times 5}^{\text{att}}+{\text{R}}_{5\times 5}$$

The residual-enhanced feature maps are then concatenated across channels to form a unified representation in Equation 6:

(6) $$\text{concat}\_\text{out}=\text{Concatenate}([{\text{R}}_{1\times 1},{\text{R}}_{3\times 3},{\text{R}}_{5\times 5}])$$

Finally, as shown in Equation 7, dimensionality reduction and feature fusion are performed by first concatenating the outputs, followed by a standard convolution operation, a depthwise separable convolution, and a concluding 1×1 projection layer.

(7) $${\text{X}}_{\text{out}}=\text{Conv }(\text{Depthwise Conv }(\text{Conv }(\text{Conv }(\text{Concat}\_\text{out}))))$$

Here, X_out_ denotes the final output of the MRADB. The MRADB design has the potential to assist test networks in acquiring features that are both enhanced and efficient due to multi-scale attention. In particular, the module is suitable for doing tasks that require high rates of vision, like medical image segmentation, object detection, and classification.

#### 2.2.3 Convolution block attention module (CBAM)

CBAM in UlcerNet utilizes spatial attention and a sequential channel to compute feature map recalibration. The channel attention module achieves salience feature highlighting by taking advantage of inter-channel dependencies, where the spatial attention module concentrates on the relevant parts of the spatial pattern by pooling the information along the channel axis (20). The combination significantly enhances feature representation compared to using either attention mechanism independently.

(8) $${\text{F}}_{\text{out}}={\text{h}}_{\text{attn}}({\text{Z}}_{\text{coord}})$$

In Equation 8, F_out_ denotes the output feature map after applying the attention refinement function h_attn_() to the coordinate-enhanced feature map Z_coord_. The function h_attn_() recalibrates features by emphasizing informative regions while suppressing irrelevant ones.

**Algorithm 1** outlines an attention-guided dense convolutional network for segmenting DFUs from input images of size 384×384×3. Initially, coordinate information is embedded into the input using a coordinate convolution layer. After that, patch folding is considered to reduce spatial dimensions and increase channel depth. A sequence of residual modules (MRADB blocks) and max pooling layers gradually extracts high-level features through the encoder. A multi-receptive field bottleneck (MRFB) then captures global context. The decoder reconstructs spatial details via upsampling and skip connections, then concatenates them with the encoder features. CBAM is applied to enhance relevant features. Finally, the outcome is unfolded to the input resolution in order to produce a segmentation map of size 384×384×2.

Algorithm 1

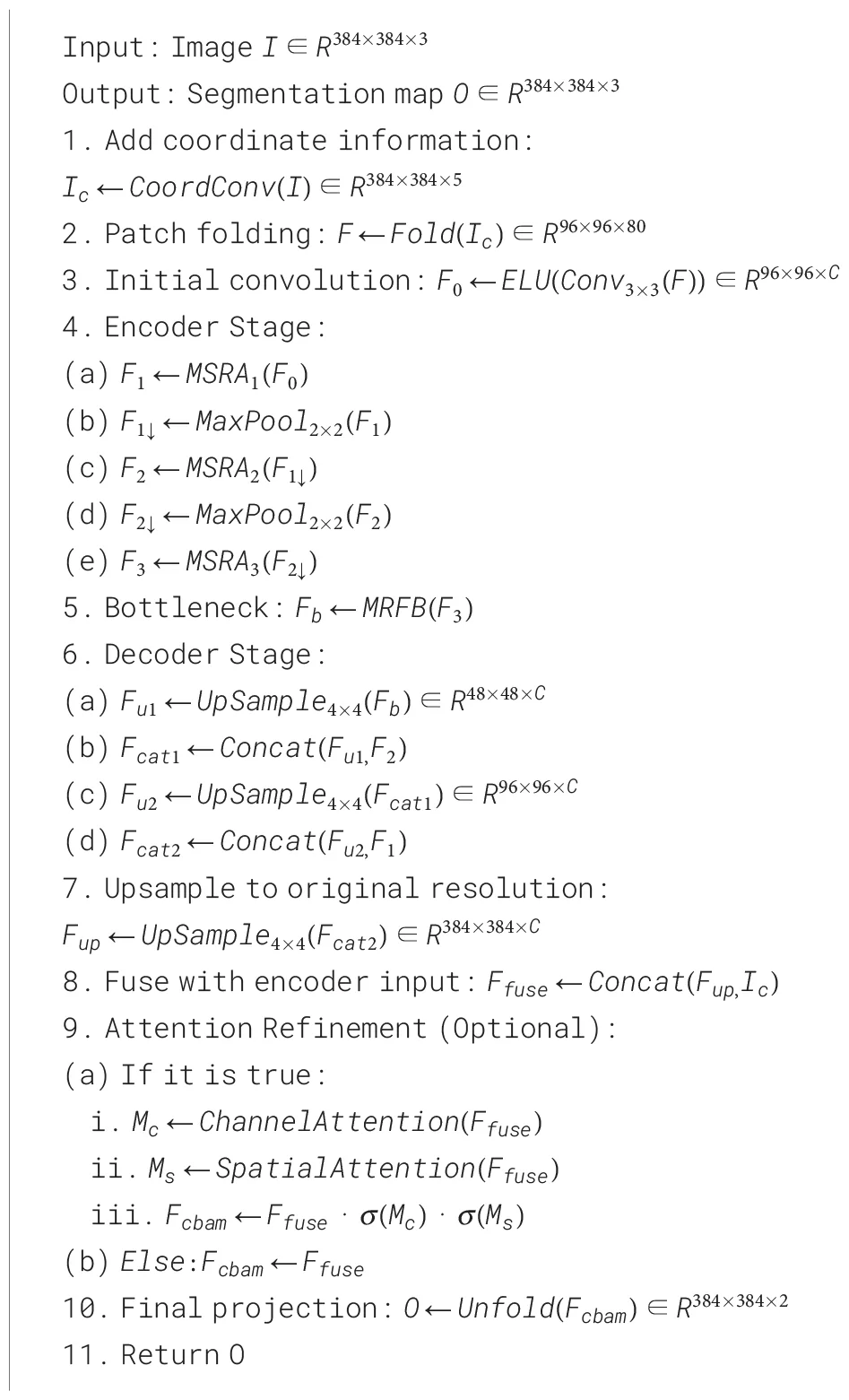

#### 2.2.4 Loss function

A composite function is used to increase the segmentation quality of the pixel using dice loss and cross-entropy. Cross-entropy concentrates on the errors made by the individual pixel by imposing a penalty for incorrect classification. On the other hand, dice loss aids in resolving the problem of class imbalance because it assigns more significance to the overlay between the ground truth and predicted areas.

(9) $$\text{L}={\text{λ}}_{1}{\text{L}}_{\text{dice}}+{\text{λ}}_{2}{\text{L}}_{\text{ce}}$$

(10) $${\text{L}}_{\text{dice}}=1-\frac{2\sum {\text{Y}}_{\text{pred}}{\text{Y}}_{\text{true}}+\text{δ}}{\sum {\text{Y}}_{\text{pred}}+\sum {\text{Y}}_{\text{true}}+\text{δ}}$$

(11) $${\text{L}}_{\text{ce}}=\frac{1}{\text{M}}\sum \left[{\text{Y}}_{\text{true}}\text{log}{\text{Y}}_{\text{pred}}+(1-{\text{Y}}_{\text{true}})\text{log}(1-{\text{Y}}_{\text{pred}})\right]$$

Equation 9 defines the hybrid loss *L* as a combined weighted dice loss L_dice_ and cross-entropy loss L_ce_, balanced by coefficients λ_1_ and λ_2_. As shown in Equation 10, L_dice_ evaluates the overlay between the actual area Y_true_ and the predicted mask Y_pred_, with a small constant added for stability. Equation 11 defines L_ce_, the pixel-wise cross-entropy loss averaged over M pixels, penalizing misclassification of individual pixels.

## 3 Results analysis

In this section, a complete test of the proposed model is carried out using various quantitative and qualitative experiments. Data augmentation is first explained, then the hyperparameters and the settings used for the training are explained. Followed by the performance evaluation metrics used are added. The effectiveness of the proposed method is then assessed by experimental results and comparisons with the state-of-the-art methods. An ablation study is done to study the contribution of individual architectural components towards performance. Finally, the efficacy, robustness, limitations, and clinical applicability of the results to automated diabetic foot ulcer segmentation are discussed.

### 3.1 Data augmentation

Data augmentation is used to augment data to enhance model generalizability. The augmentations consist of random horizontal and vertical flips and rotations. This creates diversity in the training data and approximates varied imaging conditions that can allow the model to be more generalized to unobserved samples.

(12) $${\text{χ}}_{\text{aug}}={\text{T}}_{\text{flip}}\circ {\text{T}}_{\text{rotate}}(\text{χ})$$

Where *X*_aug_ is the augmented image produced by successively rotating and flipping the original image *X* in Equation 12. This information addition increases model generalization by adding spatial orientation variation.

### 3.2 Training hyperparameters

The UlcerNet Model was trained by tuning hyperparameters in a proper manner, as given in **Table 3**. The model was trained with the Adam optimizer, which adapts learning rates with an initial value of 0.001. To increase convergence and decrease overfitting, the learning rate was decreased to 10% of its current rate for every 10 epochs when validation loss did not improve. The mini-batch size was chosen as 4, adequate enough to keep GPU memory and throughput in check while providing stable behavior during training. The dataset was trained for 250 epochs to capture the intricate patterns of the dataset efficiently through the model.

**Table 3:** Training hyperparameters for the UlcerNet model.

Hyperparameter	Value
Optimizer	Adam
Learning Rate Initialization	0.001
Schedule of Learning Rate	Reduced to 10% every 10 epochs (if no validation improvement)
Batch Size	4
Total Epochs	250
GPU Used	NVIDIA GeForce RTX 2070
Input Image Size	384 × 384 × 3
Output Tensor Size	384 × 384 × 2

**Figure 5** displays the chart of UlcerNet Model training and validation over 100 epochs. **Figure 5a** shows that the dice coefficient rises quickly and remains high for both training and validation sets. This leads to robust and accurate segmentation performance. **Figure 5b** illustrates a rapid decrease in loss for both training and validation sets. This stabilizes the loss at a low value around 0.4 and suggests effective model learning and convergence. Thus, the UlcerNet model has been well trained and performed well for its intended task based on these performance outcomes.

**Figure 5:**
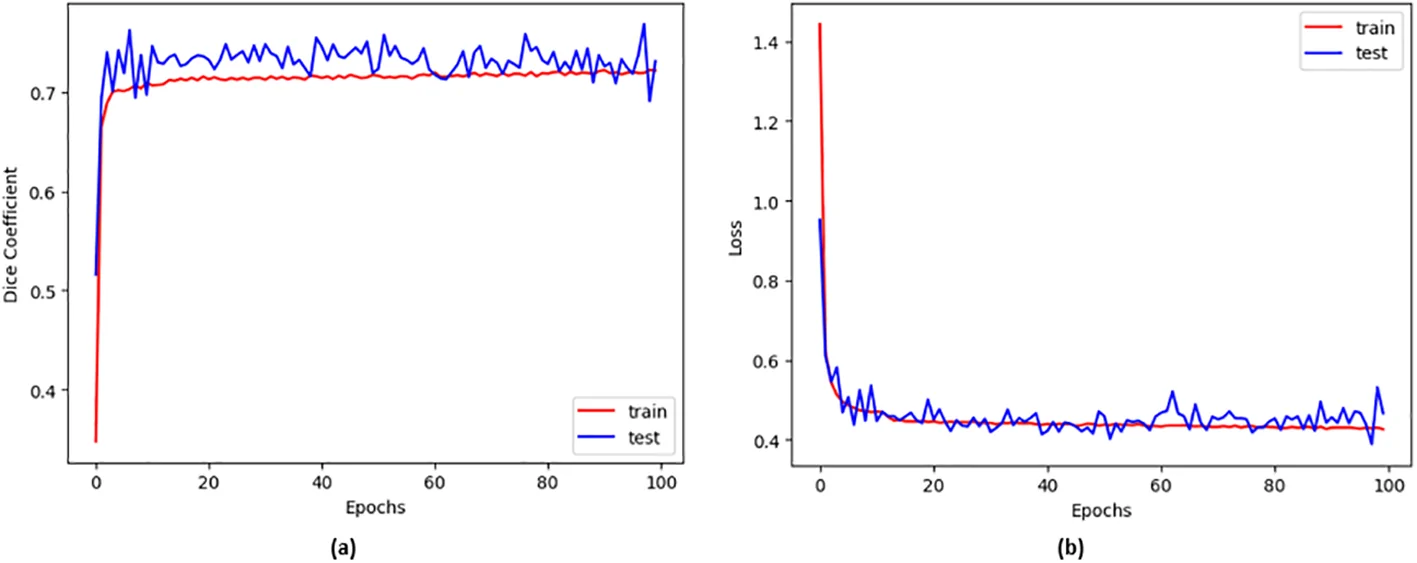
UlcerNet model training and validation curves over epochs: **(a)** dice coefficient and **(b)** loss.

### 3.3 Evaluation metrics

The regions of overlay can be assessed using the Dice coefficient and Intersection over Union (IoU), and also used to assess the accuracy of the segmentation prediction.

(13) $$\text{Dice}=\frac{2\left|{\text{Y}}_{\text{true}}\cap {\text{Y}}_{\text{final}}\right|}{\left|{\text{Y}}_{\text{true}}\right|+\left|{\text{Y}}_{\text{final}}\right|},\text{IoU}=\frac{\left|{\text{Y}}_{\text{true}}\cap {\text{Y}}_{\text{final}}\right|}{\left|{\text{Y}}_{\text{true}}\cup {\text{Y}}_{\text{final}}\right|}.$$

The Dice correlation and IoU measures are defined by Equation 13. This metric evaluates the region-wise likeness between the ground truth Y_true_ and the predicted mask Y_final_ by computing the IoU, defined as the proportion of the overlapping region to the combined region of both regions. The IoU metric is a secondary metric for assessing segmentation that makes use of both the actual region and the predicted region.

### 3.4 Side output layer results

As given in **Table 4**, the findings of the UlcerNet were compared with the DFUC 2022 dataset across different side output layers. This helps to assess the contribution of each of the output layers. The fused output layer of UlcerNet achieved a dice coefficient value of 0.735 and an mIoU of 0.76047. This establishes the model’s robust segmentation capability while aggregating features from different layers. x1 (side output layer) yielded a dice coefficient of 0.7311 and mIoU of 0.76105, indicating comparable performance to the fused layer. The performance slightly declined in deeper side outputs: x2 (side output layer) reached a dice coefficient of 0.72496 and mIoU of 0.75886; x3 (side output layer) recorded 0.71944 and 0.75434; and x4 (side output layer) yielded 0.72 and 0.74575, respectively.

**Table 4:** UlcerNet model outcomes for side output layers.

Model type	Dice score	mIoU
Fused Output	0.7350	0.76047
x1	0.7311	0.76105
x2	0.72496	0.75886
x3	0.71944	0.75434
x4	0.7200	0.74575

x1, x2, x3, and x4 denote the side output layers.

These results validate the contribution of all side output layers, and the fused layer is effectively integrated by integrating multi-scale features for optimal segmentation performance. The Dice and mIoU performance of UlcerNet on side output layers is shown in **Figure 6**. It highlights the segmentation effectiveness on the DFU dataset across different layers.

**Figure 6:**
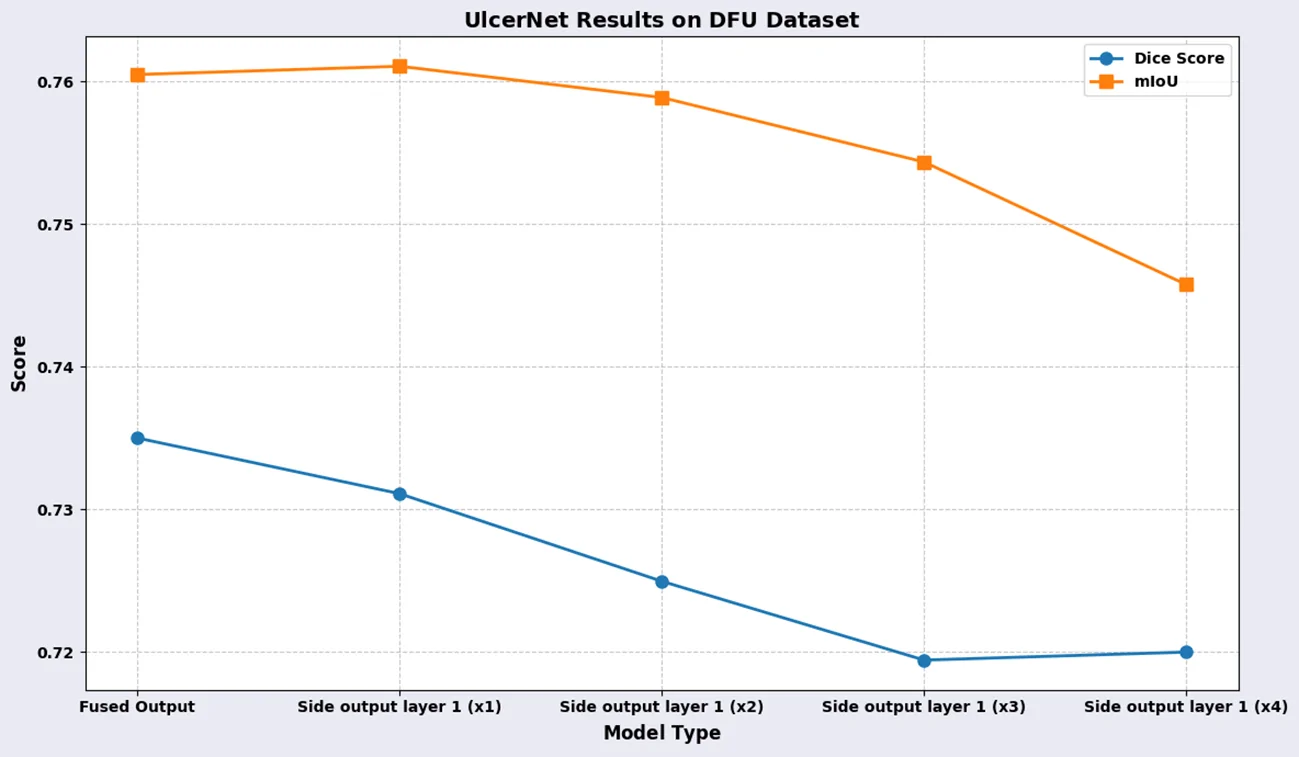
Dice coefficient and mIoU results of UlcerNet on side output layers.

### 3.5 Ablation study results

In UlcerNet, an ablation study to understand the contribution of the coordinate convolution layer and the global attention mechanism alone and in combination was carried out and shown in **Table 5**. The highest performance of the model was obtained when the coordinate layer was added, as well as the global attention, with a dice coefficient of 0.735 and an mIoU of 0.76047. The entity of disabling the coordinate layer and keeping global attention resulted in the dice score decreasing sharply to 0.6291, even though mIoU was quite high (0.7586).

**Table 5:** Results of various ablation studies conducted on the DFU dataset.

Model variant	Coordinate conv	MRADB	MRFB	CBAM	Dice score	mIoU
Baseline Encoder-Decoder	–	–	–	–	0.6010	0.6284
+ Coordinate Conv	✓	–	–	–	0.6120	0.7010
+ MRADB	✓	✓	–	–	0.6291	0.7233
+ MRFB	✓	✓	✓	–	0.6291	0.6291
+ CBAM (Proposed Work)	✓	✓	✓	✓	**0.**7350	**0.**76047

On the other hand, the proposed work with a kept coordinate layer and removed global attention reached a Dice coefficient of 0.7112 and mIoU of 0.7233, which demonstrated the significance of attention mechanisms in spatial consistency. The lowest performance was in both components, with the best Dice coefficient of 0.612 and mIoU of 0.701. The above results provide strong support for the importance of the coordinate information and mechanisms of attention in refining the segmentation accuracy and robustness of the UlcerNet model. **Figure 7** shows the ablation study results that indicate the Dice and mIoU metric values on the DFU dataset.

**Figure 7:**
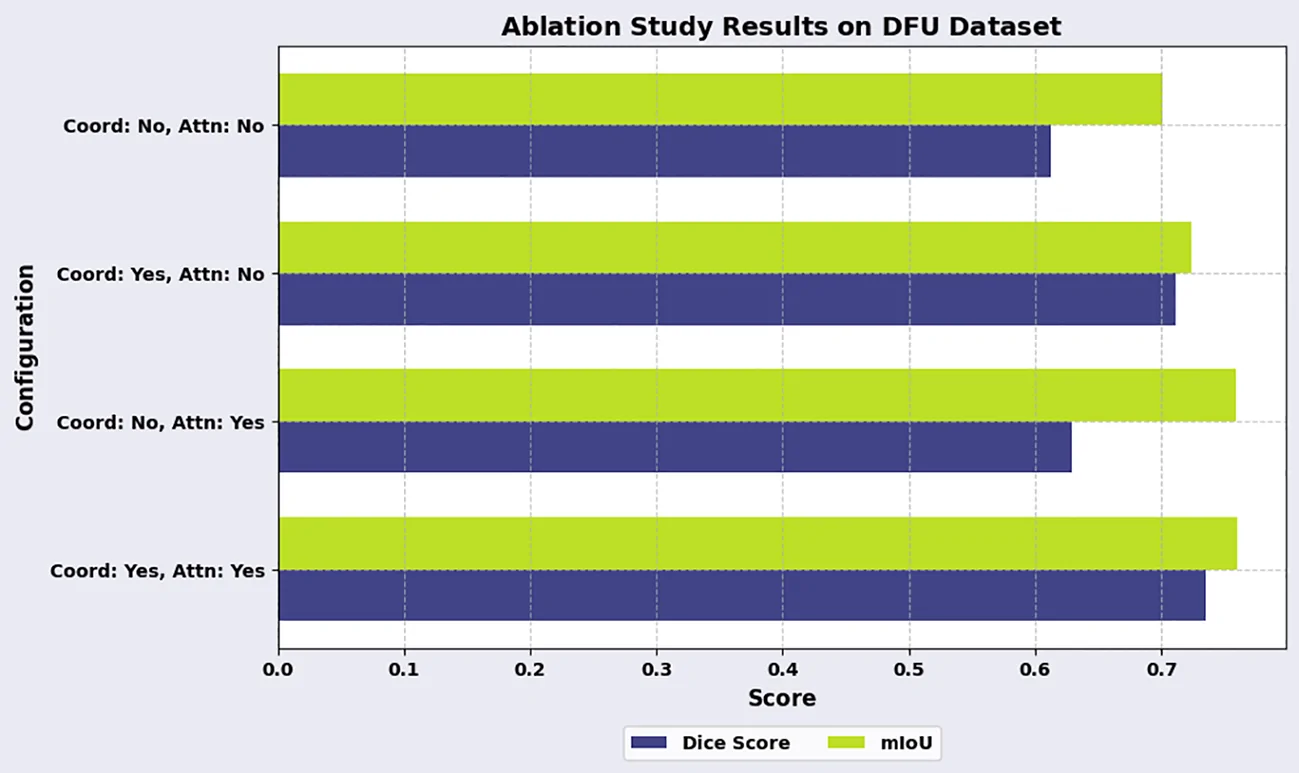
Ablation study: Dice and mIoU on DFU dataset.

### 3.6 Statistical significance analysis

A paired non-parametric analysis was performed to assess the statistical significance of the performance gain of UlcerNet over a true baseline using the same test set of 379 images. Baseline was the ablation-study Baseline Encoder-Decoder from **Table 5**, first row, reimplemented as a plain U-Net with the same channel widths, and trained with the same data split, augmentation, loss function, optimizer and training protocol as UlcerNet. Three independent random seeds were used for each model. For the primary comparison, image-by-image two-sided Wilcoxon signed rank test was used (n=379) to compare UlcerNet seed 2 with baseline seed 2. The 10,000 bootstrap resamples were used to construct a 95% confidence interval (CI) for the median paired difference.

**Table 6** displays the primary comparison that showed statistically significant improvements for UlcerNet in both metrics. For Dice, UlcerNet achieved a mean score of 0.7459 compared with 0.6892 for the baseline, with a median paired improvement of 0.0209 (95% CI: 0.0119–0.0336), Wilcoxon W = 19,700, and p=2.17×10^−14^. For IoU, the corresponding means were 0.6417 and 0.5783, with a median paired improvement of 0.0290 (95% CI: 0.0184–0.0488), W = 19,364, and p=6.32×10^−15^. Thus, both performance gains are statistically significant (p<0.001), and the confidence intervals (95%) exclude zero. The statistical test uses the per-image outputs from the seed-2 best runs; the manuscript’s aggregate UlcerNet result reported elsewhere remains unchanged.

**Table 6:** The analysis of statistical significance of UlcerNet and matched baseline.

Metric	UlcerNet mean	Baseline mean	Median paired difference	95% CI	Wilcoxon W	P-value
Dice	0.7459	0.6892	+0.0209	[0.0119, 0.0336]	19,700	2.17×10^−14^
IoU	0.6417	0.5783	+0.0290	[0.0184, 0.0488]	19,364	6.32×10^−15^

**Table 7** contains all nine UlcerNet–baseline seed-pair combinations and were also evaluated in order to determine their robustness to random initialization. For both Dice and IoU, all pairings resulted in p<1×10^−10^, meaning the observed improvement was unlikely to be attributable to any one favorable pairing of seeds.

**Table 7:** Wilcoxon signed-rank p-values across all nine seed-pair combinations.

Seed pairing	Dice p-value	IoU p-value
U1 vs B1	2.81×10^−13^	8.49×10^−14^
U1 vs B2	3.30×10^−12^	1.93×10^−12^
U1 vs B3	4.40×10^−13^	2.54×10^−13^
U2 vs B1	1.04×10^−15^	1.16×10^−16^
U2 vs B2	2.17×10^−14^	6.32×10^−15^
U2 vs B3	4.44×10^−15^	9.98×10^−16^
U3 vs B1	8.48×10^−13^	3.78×10^−13^
U3 vs B2	5.43×10^−11^	4.61×10^−11^
U3 vs B3	2.39×10^−11^	1.94×10^−11^

U1–U3 are the three separate runs of UlcerNet, while B1–B3 are the three separate baseline runs. The comparison that the headline is talking about is U2 vs B2. The principal analysis is a one matched baseline, so there is no need for a multiple comparison correction to the headline test. A robustness check (nine-pair analysis) is provided.

The model was trained from scratch with three different random seeds, the same training/testing split (1,620/379 images) and identical hyperparameters to assess the reproducibility and training stability of UlcerNet. All runs were done with a batch size of 4, accumulated in two steps (effective batch size = 8), a number of training epochs of 150, and an optimizer of the Adam type with a learning rate of 0.001. Only the weight initialization and shuffling of the data was affected by the random seeds. The best validation Dice in each run was saved and per-image performance scores were finally calculated on a test set of 379 images.

**Table 8** defined that the proposed UlcerNet framework is stable and reproducible, as the relatively small standard deviations across the three independent runs (Dice: 0.0057; IoU: 0.0070) indicate. The mean performance also matches the single-run performance reported earlier, indicating the model’s stability over various random starting conditions and shuffling of the data, a necessary condition for the model’s usefulness. The reproducibility results are also presented as Dice = 0.7397 ± 0.0057 and IoU = 0.6339 ± 0.0070 (mean ± SD, across three different trainings).

**Table 8:** Performance stability of UlcerNet across three independent training runs with different random seeds.

Run	Dice	IoU	Precision	Recall	Specificity	Best Epoch
Seed 1	0.7384	0.6313	0.7257	0.8277	0.9943	128
Seed 2	0.7459	0.6417	0.7673	0.7890	0.9954	147
Seed 3	0.7348	0.6285	0.7238	0.8156	0.9949	132
Mean ± SD	0.7397 ± 0.0057	0.6339 ± 0.0070	0.7389 ± 0.0246	0.8108 ± 0.0198	0.9949 ± 0.0005	—
Minimum	0.7348	0.6285	0.7238	0.7890	0.9943	—
Maximum	0.7459	0.6417	0.7673	0.8277	0.9954	—

### 3.7 Comparison with benchmark models

As it is clearly seen from **Table 9**, it compares the proposed UlcerNet with existing deep learning segmentation networks published from 2015 to 2025 on the DFUC 2022 benchmark. The comparison is performed using metrics commonly used in segmentation, namely IoU, Dice coefficient, accuracy, precision, and recall. The proposed UlcerNet demonstrates superlative overall segmentation performance, especially in the Dice coefficient of 0.7350, and competitive performance in further evaluation metrics.

**Table 9:** A quantitative evaluation of UlcerNet compared to state-of-the-art models on the DFUC 2022 dataset.

Models	Year	IoU	Dice	Acc	Prec	Rec
FCN (21)	2015	0.5995	0.6822	0.4128	0.7131	0.5673
U-Net++ (22)	2018	0.5500	0.6229	0.4384	0.7446	0.5086
PSPNet (23)	2019	0.5705	0.6482	0.5489	0.6549	0.5518
TransUNET (24)	2021	0.4172	0.4261	0.4430	0.8348	0.4377
HarDNet-DFUS (25)	2023	0.7287	0.5981	N/A	N/A	N/A
Attention UNet (26)	2024	0.7140	0.6511	N/A	N/A	N/A
UFOS-Net (27)	2025	0.6134	0.7309	0.6952	0.7929	0.5942
UlcerNet (Proposed model)	2026	0.7294	0.7350	0.7130	0.8241	0.6428

The proposed model, UlcerNet, has been extensively assessed against other established deep learning models on the DFUC 2022 dataset, as shown in **Table 10**. The findings indicate that UlcerNet was better than all the benchmark models associated with Dice Score and mean IoU, which emphasizes its better segmentation performance. Specifically, UlcerNet obtained a Dice coefficient of 0.735 and mIoU of 0.76047, surpassing the execution of DeepLabv3+ (28) (Dice 0.6277, mIoU 0.5224). Among the fully convolutional networks, FCN8 with ResNet50 (29) backbone yielded a Dice coefficient of 0.4993 and mIoU of 0.3963, while FCN8 with VGG (30) achieved 0.5101 and 0.3952, respectively. FCN32 with ResNet50 and VGG attained dice coefficients of 0.4334 and 0.5708, and mIoUs of 0.3372 and 0.4549. SegNet (31) integrated with ResNet50 attained a dice coefficient of 0.4768 and an mIoU of 0.3676, while its VGG-based counterpart recorded slightly lower values of 0.4596 and 0.3469, respectively.

**Table 10:** Comparison of dice score and mIoU values of different deep learning models on the DFU dataset.

Model	Backbone	Dice score	mIoU
FCN8	ResNet50	0.4993	0.3963
FCN8	VGG	0.5101	0.3952
FCN32	ResNet50	0.4334	0.3372
FCN32	VGG	0.5708	0.4549
SegNet	ResNet50	0.4768	0.3676
SegNet	VGG	0.4596	0.3469
U-Net	ResNet50	0.0646	0.0371
U-Net	VGG	0.1446	0.0878
DeepLabV3+	ResNet50	0.6277	0.5224
UlcerNet (Proposed model)	MRADB	0.7350	0.76047

Among all assessed models, U-Net Versions provided the most disappointing results with the Dice Scores of 0.0646 (ResNet50) and 0.1446 (VGG), and mIoUs of 0.0371 and 0.0878. UlcerNet whose dice score on the official test set of 0.7287. The manuscript avoids discussing the most desirable solution, which artificially exaggerates the perceived novelty and performance of UlcerNet. Such results highlight the best performance of UlcerNet in categorizing diabetic foot ulcers accurately and consistently, and being better than other modern models. **Figure 8** shows a visual comparison between Dice score and mIoU statistics of various models on the DFU dataset, which effectively shows the superiority of UlcerNet to the baseline architectures.

**Figure 8:**
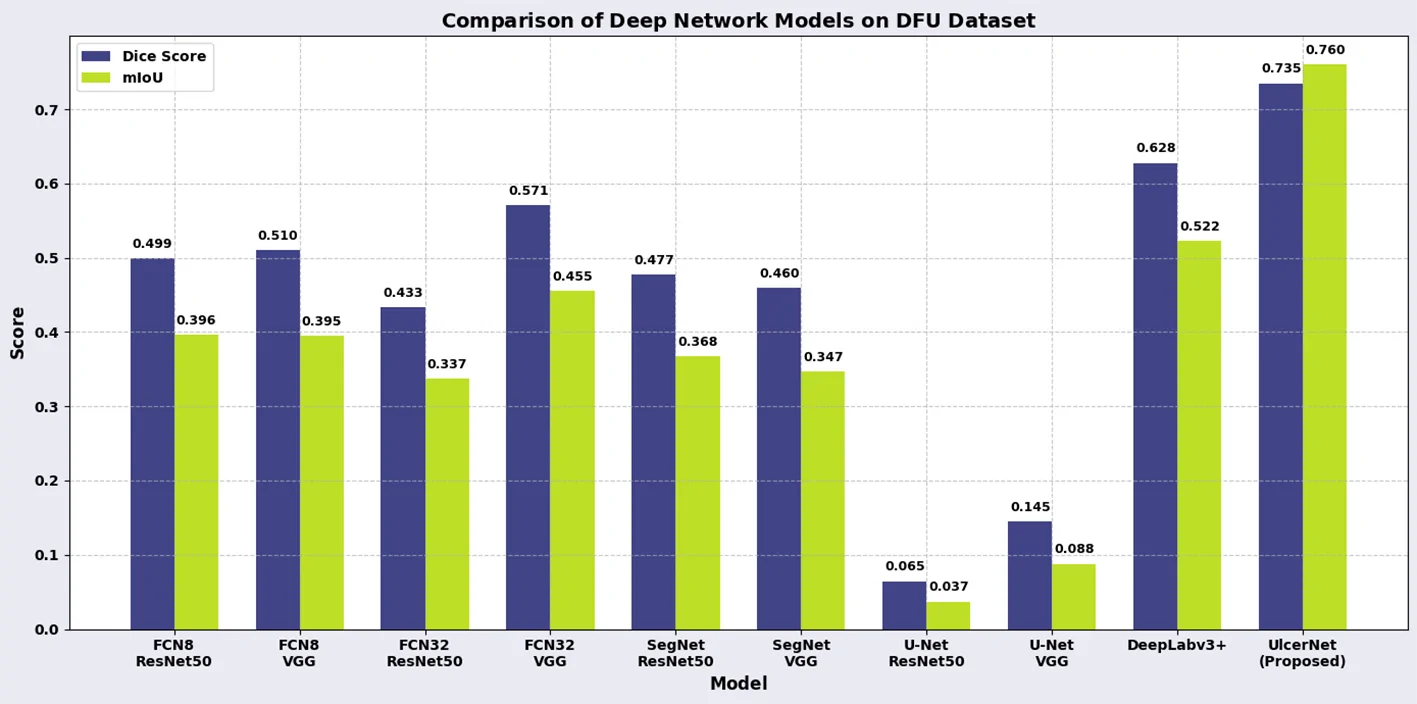
Dice and mIoU comparison of models on the DFU dataset.

### 3.8 Computational efficiency and model complexity

The computational efficiency and feasibility of deployment of UlcerNet were assessed in **Table 11** by measuring the complexity of the model and its performance in making inferences on an NVIDIA GeForce RTX 4070 SUPER. The model has 7,823,736 trainable parameters (≈7.82 million) and a checkpoint size of 30.27 MB. The computational requirement for an input resolution of 384×384×3 is 158.12 GFLOPs, which is 2×MACs.

**Table 11:** Computational complexity and inference efficiency of UlcerNet.

Metric	Value
Total parameters	7,823,736 (≈7.82 M)
Model checkpoint size	30.27 MB
Input resolution	384 × 384 × 3
GFLOPs (2×MACs)	158.12
Inference time (batch = 1, FP32)	68.59 ms/image
Throughput (batch = 1, FP32)	14.6 FPS
Inference time (batch = 8, FP32)	34.21 ms/image
Throughput (batch = 8, FP32)	29.2 FPS
Measurement device	NVIDIA GeForce RTX 4070 SUPER

The batch-1 measurements represent the standard single-image inference protocol. Batch-8 measurements are provided as an additional throughput reference.

When it comes to measuring the performance of UlcerNet on a single image with FP32 precision and batch size 1, it took 68.59ms per image, or 14.6 frames per second (FPS). With a batch size of 8, the average inference time reduced to 34.21ms per image and was able to achieve 29.2 FPS.

With these results, we can conclude that UlcerNet is an intermediate size model with efficient inference performance for the images used in this study. The single-image inference time of 68.59ms indicates that the model can perform inference on individual DFU images within a fraction of a second on the evaluated GPU. But in real-time clinical deployment, the speed of inference isn’t the only factor to consider, it also relies on availability of hardware, time required for image capture and preprocessing, post processing, and integration with the clinical workflow. Thus, the results reported do not prove real-time deployment in the clinical setting, but computational feasibility.

## 4 Discussion

The proposed model of UlcerNet is effective in the segmentation of DFUs, as it is demonstrated by the experimental results. It has superior accuracy and robustness related to the already existing models. The highest Dice coefficient achieved by this model is 0.735, and mIoU is 0.76047 in the case of a fused output layer, indicating that the fused output mask is highly correlated with the ground truth mask. Competitive results were also achieved using side output layers, whose Dice coefficient ranged between 0.72 and 0.7311 and mIoUs between 0.74575 and 0.76105, with the difference in scale feature learning at various depths in the network. The importance of the coordinate convolution layer and global attention mechanisms in improving segmentation also received additional focus in ablation studies. Omission of these parts resulted in significant performance decreases, with the decline of the Dice score down to 0.612 and mIoU down to 0.701. Therefore, justify their presence in the architecture.

Comparative evaluations of UlcerNet with benchmark models like FCN8, FCN32, SegNet, UNet, and DeepLabv3+ demonstrated that UlcerNet always offers high performance as compared to the other models. It provides results in Dice Score and mIoU by considerable margins. As one example, DeepLabv3+ is the most powerful baseline model, which achieved a 0.6277 Dice score and 0.5224 mIoU. They are both significantly below the UlcerNet metric values. These results suggest that Multi-scale residual attention combined with hierarchical feature aggregation in UlcerNet resulted in its high performance. The suggested work can detect ulcers of various sizes (0.04% up to 35.05% of the image area) and forms. It validates the applicability and feasibility of UlcerNet in a clinical environment to be implemented in automated segmentation and analysis of DFU. **Figure 9** contains additional DFU images with their segmented outputs. It demonstrates the proposed model’s capability to delineate complex ulcer boundaries. It shows real DFU images along with their tumor segmentation outputs. It demonstrates the model’s capability of producing precise segmented results on clinical data.

**Figure 9:**
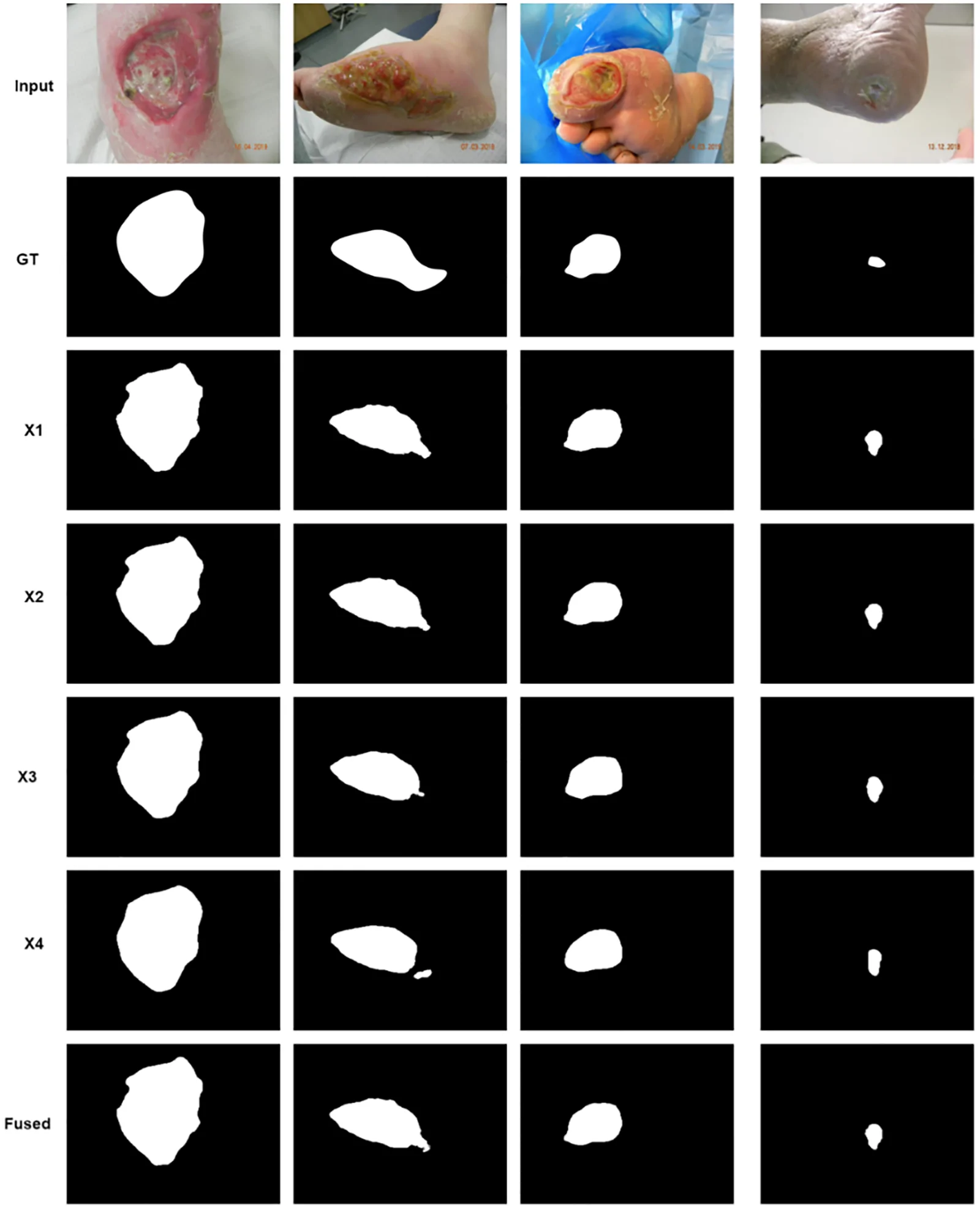
Real DFU images and their tumor-segmented output images visualization at different.

Most significantly, the statistical analysis corroborates this performance comparison. On the same 379 test images, UlcerNet significantly outperformed the matched baseline in both Dice (median paired gain +0.0209, 95% CI [0.0119, 0.0336], p=2.17×10^−14^) and IoU (median paired gain +0.0290, 95% CI [0.0184, 0.0488], p=6.32×10^−15^) using the two-sided Wilcoxon signed-rank test. This is further evidence that the observed gain is not due to random initialization, as this same significance was seen in all nine seed-pair combinations.

**Table 12** analyze the effects on segmentation performance, the 379 test images were sorted by their Dice (per image Dice, best-performing run [Seed 2]: Dice = 0.7459). Lesion area, lesion to background grayscale contrast and lesion irregularity assessed. Irregularity of the boundary was quantified as perimeter^2/4*pi*area.

**Table 12:** Correlation of lesion characteristics with per-image Dice scores on the test set.

Factor	Correlation with dice
Lesion area (%)	+0.155
Lesion-to-background contrast	+0.338
Boundary irregularity	−0.248

The Dice coefficient had the highest correlation with lesion-to-background contrast, and the boundary irregularity had the highest correlation with lesion area, suggesting that lesions with low contrast and highly irregular boundaries are harder to segment.

In **Figure 10**, two typical failure cases were detected. Case 100199 (Dice = 0.000; area = 1.82%; contrast = 2.4) had very low contrast, which resulted in severe under-segmentation. Case 101998 (Dice = 0.000; area = 0.49%; irregularity = 6.83) was a small lesion that had a very irregular boundary, leading to less accuracy in segmentation. The 12 worst failures and the qualitative analyses of them are included in the supplementary failure analysis report.

**Figure 10:**
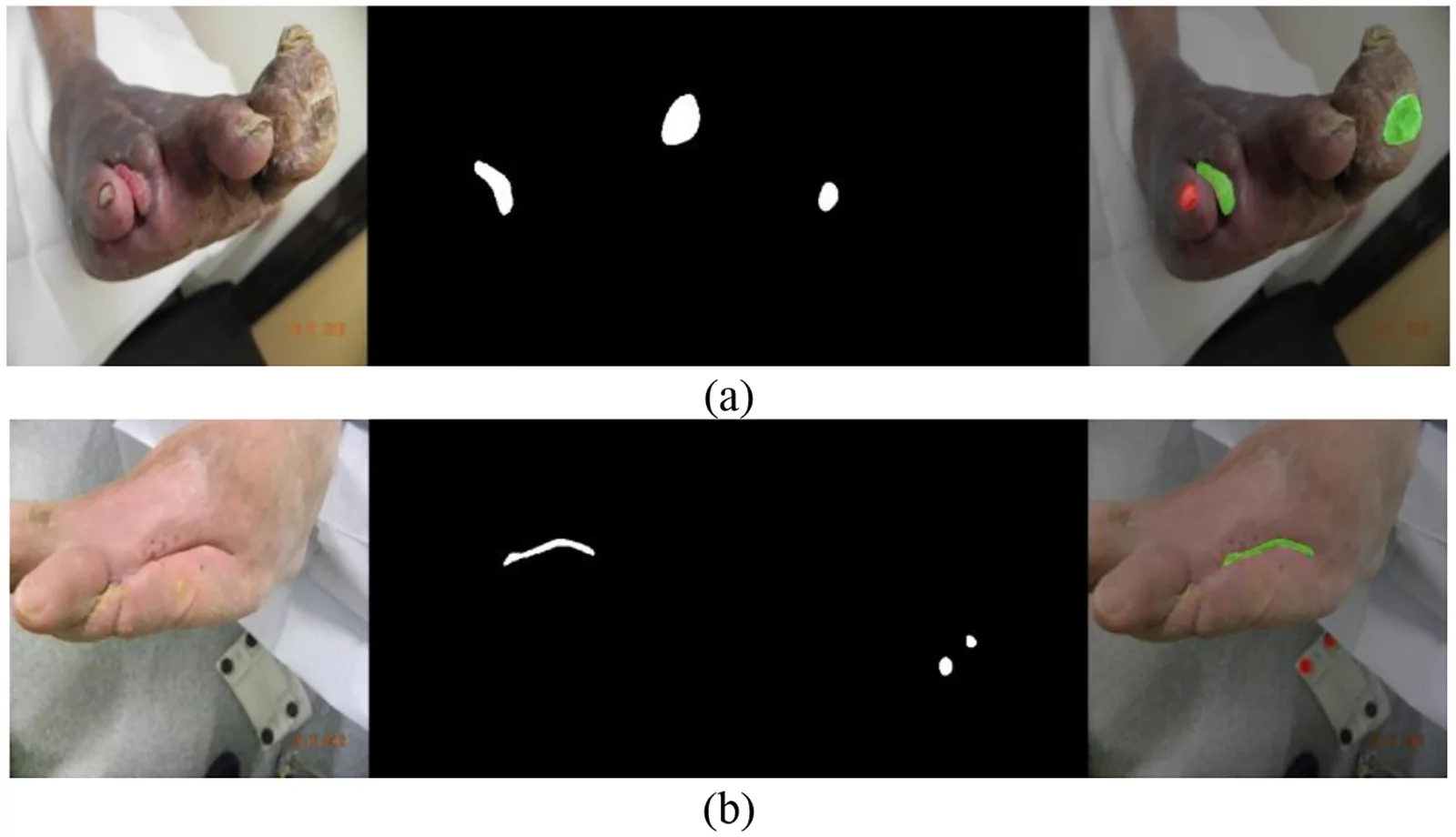
Representative failure cases **(a)** Case 100199 (Dice=0.000, area=1.82%, contrast=2.4). Very low-contrast lesion; the model under-segments almost entirely (85% of the error is false negative). **(b)** Case 101998 (Dice=0.000, area=0.49%, irregularity=6.83). A tiny lesion with a highly irregular boundary -- both risk factors compound here.

In **Figure 11**, in order to measure the interpretability of UlcerNet, two attention measures were derived during inference: Grad-CAM on the deepest encoder dense block in relation to the fused foreground probability, and the last CBAM channel and spatial attention maps calculated during the forward pass. In positive cases, both the Grad-CAM and CBAM spatial attention maps mostly concentrate on the ulcer area and its boundaries, showing that the irrelevant areas have been successfully suppressed. However, in negative cases, the attention maps appear weaker and more dispersed, showing lower confidence in the detection of the hard-to-find low-contrast lesions rather than consistently erroneous localization to other regions.

**Figure 11:**
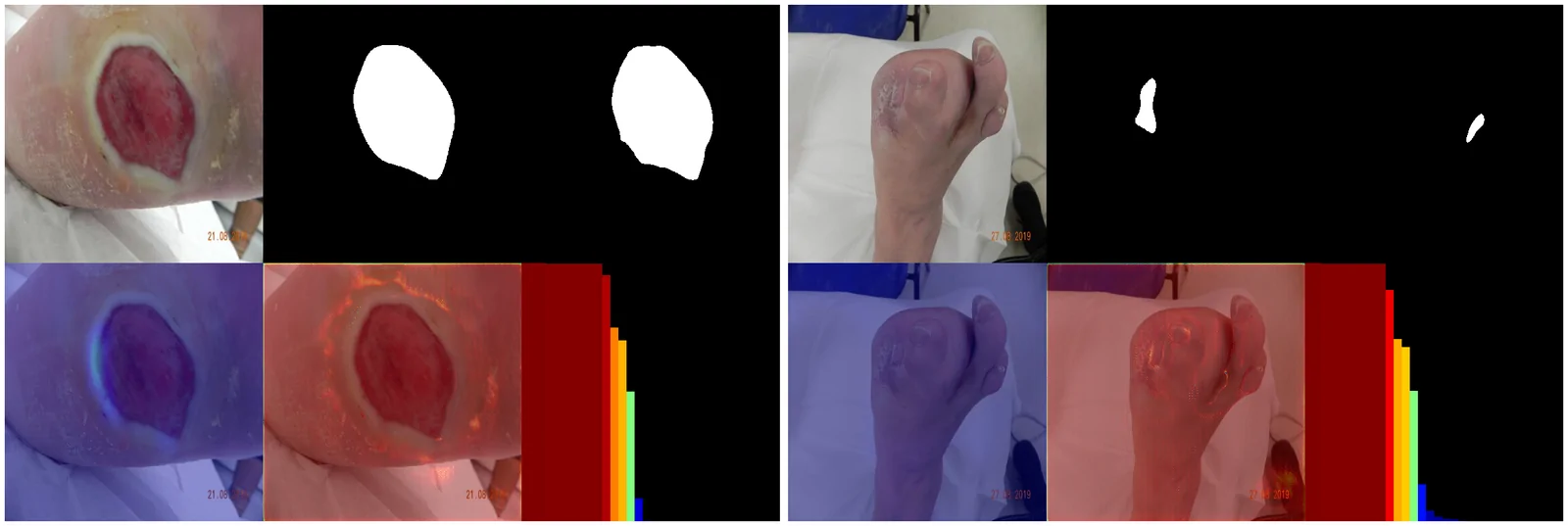
**(a)** Success Case- A correctly segmented test image. Top row: input, ground truth and prediction. Bottom row: Grad-CAM (rings the ulcer boundary), CBAM spatial-attention gate and CBAM channel-attention weights (top channels by activation strength). **(b)** Failure Case- A missed-detection test image (also in the failure-case list above). Grad-CAM activation is weak and diffuse rather than sharply localized, visually explaining the under-segmentation.

There are several limitations associated with the performance of UlcerNet, despite its good results in the task of segmentation. First, UlcerNet was tested only in relation to the DFUC 2022 dataset, and there was no independent cross-validation of the method. Secondly, TransUNet was used for comparison with UlcerNet, but some other recently proposed architectures, like Swin-UNet and MedNeXt, were not considered within this experimental setup. In addition, the robustness of UlcerNet to variations in illumination, skin color, cameras, image quality, and other acquisition parameters was not studied systematically. Finally, the clinical validation, the expert validation, and the integration into the practice of telemedicine and clinics were beyond the scope of this work. The future directions include testing of UlcerNet in independent studies, comparison of UlcerNet with the more recent architectures for segmentation, study of robustness, expert evaluation, and the optimization for practical use.

## 5 Conclusion

This paper proposed an UlcerNet model, which is a new multi-scale residual attention dense neural network model for ulcer segmentation. It has evolved to segment diabetic foot ulcers automatically, which is an essential measure in diabetic wound management. UlcerNet was also able to solve problems like the small size of lesions, irregular shapes of ulcers, and low-contrast boundaries. The proposed UlcerNet model relieved these issues by incorporating dense convolutional blocks, hierarchical attention mechanisms, and coordinate convolution layers into the model. The presented model has demonstrated its efficiency in large-scale tests that were done on the DFU Challenge 2022. The proposed UlcerNet achieved a dice coefficient of 0.735 and an mIoU of 0.76047, which is better than the state-of-the-art models. In addition, ablation tests supported the criticality of the coordination layer and global attention layer. The degradation of performance was observed when these layers were removed, with dice and mIoU of 0.612 and 0.701, respectively. The results emphasized the ability of the proposed model to propose accurate and effective DFU segmentation. It showed good incorporation of a digital decision-support system that assists in early diagnosis and treatment planning. The next improvement will focus on testing the proposed model with other benchmark datasets. In addition, it needs to improve its flexibility to different image conditions in practice in the clinical environment.

## Data Availability

The original contributions presented in the study are included in the article/supplementary material. Further inquiries can be directed to the corresponding author.
